# The effect of dietary resistant starch type 2 on the microbiota and markers of gut inflammation in rural Malawi children

**DOI:** 10.1186/s40168-015-0102-9

**Published:** 2015-09-03

**Authors:** M. Isabel Ordiz, Thaddaeus D. May, Kathie Mihindukulasuriya, John Martin, Jan Crowley, Phillip I. Tarr, Kelsey Ryan, Elissa Mortimer, Geetha Gopalsamy, Ken Maleta, Makedonka Mitreva, Graeme Young, Mark J. Manary

**Affiliations:** Department of Pediatrics, Washington University, St. Louis, MO 63110 USA; Department of Pediatrics, Baylor College of Medicine, Houston, TX 77030 USA; The Genome Institute, Washington University, St. Louis, MO 63110 USA; Department of Medicine, Washington University, St. Louis, MO 63110 USA; NIH/NIGMS Biomedical Mass Spectrometry, Washington University, St. Louis, MO 63110 USA; Flinders Centre for Innovation in Cancer, Adelaide, Australia; Department of Community Health, College of Medicine, Blantyre, Malawi; Washington University, School of Medicine, St. Louis, MO 63110 USA

**Keywords:** High amylose maize starch, Microbiome, Short-chain fatty acids, Metabolome

## Abstract

**Background:**

Resistant starch (RS) decreases intestinal inflammation in some settings. We tested the hypothesis that gut inflammation will be reduced with dietary supplementation with RS in rural Malawian children. Eighteen stunted 3–5-year-old children were supplemented with 8.5 g/day of RS type 2 for 4 weeks. The fecal samples were analyzed for the microbiota, the microbiome, short chain fatty acids, metabolome, and proteins indicative of inflammation before and after the intervention. Subjects served as their own controls.

**Results:**

The consumption of RS changed the composition of the microbiota; at the phylum level *Actinobacteria* increased, while *Firmicutes* decreased. Among the most prevalent genera, *Lactobacillus* was increased and *Roseburia*, *Blautia*, and *Lachnospiracea incertae sedis* were decreased. The Shannon H index at the genus level decreased from 2.02 on the habitual diet and 1.76 after the introduction of RS (*P* < 0.01). Fecal acetate concentration decreased, and fecal propionate concentration increased after RS administration (−5.2 and 2.0 μmol/g, respectively). Fecal calprotectin increased from 29 ± 69 to 89 ± 49 μg/g (*P* = 0.003) after RS was given. The lipopolysaccharide biosynthesis pathway was upregulated.

**Conclusions:**

Our findings do not support the hypothesis that RS reduces gut inflammation in rural Malawian children.

**Electronic supplementary material:**

The online version of this article (doi:10.1186/s40168-015-0102-9) contains supplementary material, which is available to authorized users.

## Background

Subclinical, chronic gut inflammation, known as environmental enteric dysfunction (EED), with its associated dysbiosis has been implicated in growth faltering [1–3]. Dietary interventions to ameliorate EED must be safe, inexpensive, robust, widely available throughout the developing world, and exert reliably beneficial effects on gut health.

Resistant starch (RS) type 2 is present in cereals, tubers, legumes, and fruits [4]. In animal models, consumption of RS improves gut integrity and absorption of nutrients and reduces T cell infiltration of the mucosa [5–7]. In humans, the consumption of RS changes the composition of the microbiota and promotes the microbial fermentative production of short-chain fatty acids (SCFA), which putatively reduce gastrointestinal inflammation [8–10]. RS also improves symptoms and reduces pathology in inflammatory bowel disease [11]. In addition, RS meets the criteria for safety, durability, and availability as a dietary intervention to reduce EED. Administration of an RS during an acute attack of diarrhea reduces duration of diarrhea in adults with cholera and children [12]. These data compel an examination of RS as an agent to reduce EED.

In this pilot controlled clinical trial, we tested the hypothesis that among 3–5-year-old rural Malawian children at high risk for EED and attendant growth stunting, the introduction of RS to the daily diet for 1 month would affect changes in the microbiota and microbiome, increase the fecal content of SCFAs, and reduce markers of gut inflammation. This study was conducted in conjunction with a study of zinc homeostasis in the same population [13].

## Results

### Clinical study results

Fecal starch content was increased in all 18 subjects as they transitioned from their habitual diet to the habitual diet + RS [13]. The children avidly consumed the RS-containing donuts, and no adverse effects were noted, including increased flatulence, which is commonly reported by adults who increase dietary RS [14].

### Microbiota

**Fig. 1:**
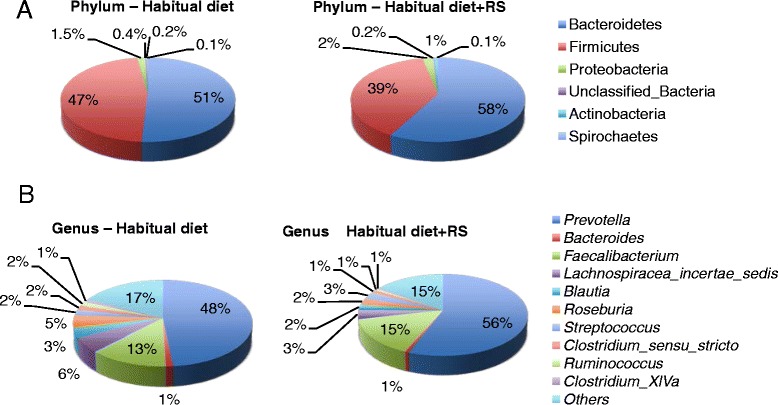
16S RNA bacterial sequences represent in fecal samples from 18 Malawian children before and after adding RS to their diet. Pie charts of average values of relative abundance (percentage of sequences) of the most abundant bacterial groups: phyla (**a**), and genus (**b**) found in the fecal microbiota

Sequences from V1–V3 and V3–V5 regions were combined to yield a more complete picture of the microbiota (Additional file 1: Table S2). Significant changes in the composition of the microbiota were seen at all phylogenetic classification levels after RS was added to the diet (Fig. 1). Notably, *Actinobacteria* increased at the phylum level, while *Firmicutes* decreased. *Coriobacteriaceae* were increased and *Lachnospiraceae* were decreased at the family level. *Lactobacillus* was increased and *Roseburia*, *Blautia*, *Lachnospiraceae Unclassified*, *Clostridium_XlVa*, *Oscillibacter*, *Butyricicoccus*, and *Lachnospiracea incertae sedis* were decreased at the genus level (Table 1).

**Table 1:** Listing of all bacterial classifications that exhibited changes in the intervention

	Habitual diet	Habitual diet + RS	*P* value
Phylum			
*Bacteroidetes*	50.5 ± 14.2	57.6 ± 11.9	0.05
*Firmicutes*	47.2 ± 13.4	39.0 ± 11.6	0.03
*Actinobacteria*	0.2 ± 0.1	0.7 ± 1.2	<0.001
Class			
*Bacteroidia*	50.6 ± 14.2	57.7 ± 11.9	0.03
*Clostridia*	42.4 ± 13.5	31.7 ± 9.2	<0.001
*Bacilli*	2.3 ± 3.2	4.3 ± 4.3	0.04
*Actinobacteria*	0.2 ± 0.1	0.7 ± 1.2	0.01
*Firmicutes* unclassified	0.1 ± 0.1	0.08 ± 0.08	0.02
Order			
*Bacteroidales*	50.7 ± 14.2	57.8 ± 11.9	0.05
*Clostridiales*	42.5 ± 13.5	31.7 ± 9.2	<0.001
*Lactobacillales*	2.3 ± 3.2	4.3 ± 4.3	0.01
*Coriobacteriales*	0.1 ± 0.1	0.7 ± 1.2	<0.001
*Bdellovibrionales*	0.1 ± 0.2	0.0 ± 0.2	0.04
*Enterobacteriales*	0.1 ± 0.3	0.8 ± 2.3	0.01
*Burkholderiales*	0.1 ± 0.1	0.1 ± 0.2	0.04
*Alphaproteobacteria*	0.0 ± 0.1	0.0 ± 0.0	0.03
*Deltaproteobacteria* unclassified	0.02 ± 0.04	0.0 ± 0.01	0.02
Family			
*Lachnospiraceae*	21.4 ± 7.4	12.2 ± 5.3	<0.001
*Bacteroidaceae*	1.2 ± 2.3	0.7 ± 1.5	0.04
*Veillonellaceae*	0.7 ± 0.7	1.2 ± 1.1	0.05
*Acidaminococcaceae*	0.6 ± 0.6	0.4 ± 0.6	0.03
*Clostridiales* unclassified	0.5 ± 0.3	0.2 ± 0.1	<0.001
*Lactobacillaceae*	0.2 ± 0.3	1.4 ± 2.6	0.04
*Coriobacteriaceae*	0.1 ± 0.1	0.7 ± 1.2	<0.001
*Eubacteriaceae*	0.1 ± 0.1	0.06 ± 0.10	0.01
*Bdellovibrionaceae*	0.1 ± 0.2	0.0 ± 0.2	0.04
*Enterobacteriaceae*	0.1 ± 0.3	0.8 ± 2.3	0.01
*Rikenellaceae*	0.1 ± 0.2	0.0 ± 0.2	0.04
Genus			
*Lachnospiracea*_*incertae*_*sedis*	5.7 ± 2.9	3.5 ± 2.2	<0.001
*Roseburia*	4.8 ± 3.2	2.6 ± 1.9	<0.001
*Lachnospiraceae* unclassified	4.6 ± 2.1	2.2 ± 1.2	<0.001
*Blautia*	3.5 ± 2.0	2.2 ± 1.6	0.03
*Bacteroides*	1.2 ± 2.3	0.7 ± 1.5	0.04
*Clostridium*_*XlVa*	1.1 ± 0.8	0.6 ± 0.5	<0.001
*Coprococcus*	0.7 ± 0.5	0.3 ± 0.2	<0.001
Oscillibacter	0.6 ± 0.9	0.3 ± 0.4	<0.001
*Butyricicoccus*	0.5 ± 0.3	0.3 ± 0.2	<0.001
*Clostridium*_*IV*	0.5 ± 0.7	0.3 ± 0.6	0.03
*Clostridium*_*XVIII*	0.4 ± 0.6	0.3 ± 0.4	0.02
Catenibacterium	0.3 ± 0.5	0.7 ± 1.0	0.01
*Lactobacillus*	0.2 ± 0.3	1.4 ± 2.6	0.04

The table shows the average values of relative abundance (percentage of sequences using both pairs) after adding resistant starch (RS) to the habitual diet of rural Malawian children. Values expressed as mean ± SD; *P* value determined by Wilcoxon Signed Ranks test

No significant changes were noted in the populations of the *Gammaproteobacteria* class (0.65 vs. 1.66 %) or the *Enterobacteriaceae* family (0.03 vs. 0.16 %), which include many of the gut microbes thought to induce a host inflammatory response. These microbes are Gram-negative bacteria, which are frequent food contaminants. These bacteria contain LPS, which induce gut inflammation in a mouse model [15]. There was no significant change in the Gram-positive genus of *Enterococcus*, another frequent food contaminant, and which also induces gut inflammation [15]. The pro-inflammatory cytokines, IL-6 and IL-8, are increased in the presence of a number of gut taxa, all belonging to the phylum *Proteobacteria* [16]. We observed no significant change in the amount of *Proteobacteria* in these children after receiving the intervention. The number of reads associated with *Eubacterium rectale*, *Parabacteroides distasonis*, and *Ruminococcus bromii* were compared in feces collected on the habitual diet and after the introduction of RS, but no differences in abundance were found. The microbiota diversity at the genera level as assessed by the Shannon H index was 2.02 on the habitual diet and 1.76 after the introduction of RS (*P* < 0.01).

*Prevotella* was the most abundant genus found in these samples and increased post-intervention (Fig. 1). The gut microbiota of humans are dominated by the genus *Bacteroides*, and can be split into enterotypes, based on the amount of *Bacteroides* and whether a decrease in *Bacteroides* is accompanied by an increase in *Ruminococcus*, *Clostridiales* and *Lachnospiraceae*, or *Prevotella* [17]. The *Prevotella*-dominant enterotype is associated with a long-term diet high in carbohydrates, like the Malawi diet [18]. Other studies have shown that *Prevotella* is predominant in the gut microbiome of children from Africa, because of the higher fiber content of their diet, relative to Italian children [19].

### Microbial genomic content

**Fig. 2:**
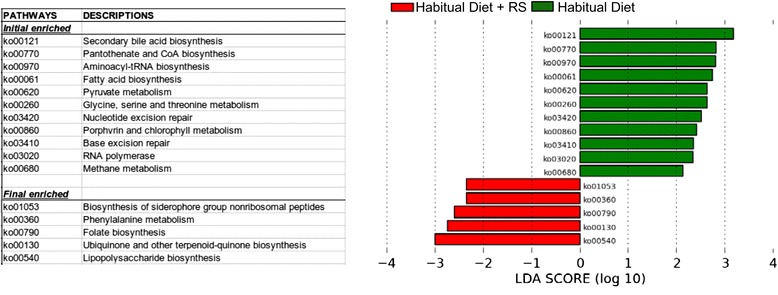
LEfse rank plot of differentially abundant pathways in gut microbiomes initial vs. final samples. LDA scores were given for different abundance of pathways before (habitual diet: *green*) and after the resistant starch was added to the habitual diet (habitual diet + RS: *red*)

Fifty-four bacterial genes encoding enzymes were found in greater abundance in the feces while on the habitual diet compared to the habitual diet + RS, while only four bacterial genes were found in greater abundance after RS was added to the diet (Additional file 1: Figure S2). In Additional file 1: Figure S2, Linear Discriminant Analysis with Effect size Galaxy server (LEfSe) results are provided for a comparison of all initial (habitual diet: green) samples vs. all final (habitual diet + RS: red) samples from individuals for which we had acceptable samples for both time points. KEGG identifiers in green are those enzymes enriched in the habitual diet samples, while those in red are enriched in habitual diet + RS samples. Larger absolute linear discriminant analyses values imply greater enrichment, and allow enriched enzymes to be ranked within each set. The most enriched enzymes in the habitual diet set are K06147 (ATP-binding cassette, subfamily B, bacterial), K02025 and K02026 (both being multiple sugar transport system permease proteins), and K00850 (6-phosphofructokinase). These enzymes are overwhelmingly associated with hits from *Eubacterium*, *Roseburia*, and *Clostridium*_*XlVa*; all bacterial species present in greater numbers in the habitual diet specimens. The most enriched enzyme in the habitual diet + RS set is K07480 (insertion element IS1 protein InsB). This enzyme is associated with the *Escherichia coli*. The most upregulated pathway with the addition of RS to the habitual diet was lipopolysaccharide biosynthesis (Fig. 2).

### Fecal SCFA and small organic molecules

In targeted gas chromatography-mass spectrometry (GC/MS) of stool, the concentration of acetate decreased with addition of RS to the diet, while propionate increased (Table 2). In GC/MS-based metabolomic profiling analyses, the aggregate population of small organic molecules after RS significantly differed from the profiles in stool before consuming the RS diet (Additional file 1: Figure S3). In particular, butyrate adducts 2-hydroxybutyrate increased 4.3-fold and 3,4 dihydroxybutanoate increased 8.4-fold (both *P* < 0.001) after RS.

**Table 2:** SCFA and metabolites with differential expression in fecal samples of 18 Malawian children fed with RS

	Habitual diet	Habitual diet + RS	*p* value
SCFA (μmol/g)			
Total short-chain fatty acids	38.0 ± 8.6	35.9 ± 9.7	0.557
Acetate	23.0 ± 6.4	17.8 ± 6.6	0.003*
Propionate	6.5 ± 2.1	8.5 ± 3.0	0.007*
Butyrate	8.5 ± 2.6	9.7 ± 4.1	0.306
	Mean ratio	95 % CI	
Metabolite			
3,4-Di-hydroxy-butanoate	4.3	[2.6, 5.9]	
β-Alanine	5.5	[1.7, 9.3]	
2-Hydroxy-butyrate	8.4	[3.6, 13.2]	
Malate	70	[1.9, 139]	
Citramalate	41	[15, 67]	
Phenylacetate	5.1	[2.0, 8.3]	

Values expressed as mean ± SD; *P* value determined by Wilcoxon Signed Ranks test. The metabolites are expressed as average of the ratio of concentration in stool after RS versus before RS administration for each child

*CI* confidence interval

*Significant difference *p* < 0.05

### Host inflammatory transcripts and proteins

Fecal calprotectin concentration increased after adding RS to the diet from 29 ± 69 to 89 ± 49 μg/g (*n* = 17, *P* = 0.003 Wilcoxon signed rank test), as well as the host messenger RNA associated with this protein (Table 3). Nine other pro-inflammatory host messages were measured in the feces while on the habitual diet and again when RS was added, with no significant changes noted (Table 3).

**Table 3:** Determination of human mRNAs in fecal samples from Malawian children before and after addition of RS

Transcript	Habitual diet	Habitual diet + RS	*P* value
Caudal type homeobox 1	0.03 ± 0.01	0.03 ± 0.01	0.424
CKLF-like MARVEL transmembrane domain containing 6	0.02 ± 0.01	0.07 ± 0.09	0.182
Family with sequence similarity 65, member B	0.01 ± 0.01	0.02 ± 0.03	0.779
Major histocompatibility complex, class II, DR alpha	0.29 ± 0.17	0.25 ± 0.14	0.332
Interleukin-1β	0.67 ± 0.73	3.56 ± 6.89	0.053
Interleukin-8	0.93 ± 0.96	2.03 ± 2.90	0.231
S100 calcium-binding protein A8 (calprotectin)	0.24 ± 0.24	1.22 ± 1.84	0.006
Sucrose isomaltase	0.01 ± 0.01	0.02 ± 0.02	0.424
Mitochondrial superoxide dismutase 2	0.65 ± 0.46	1.28 ± 1.25	0.575
Toll-like receptor-4	0.02 ± 0.01	0.09 + 0.11	0.062

The ratio of transcript: GAPDH are reported. Values expressed as mean ± SD; *p* value were determined by Wilcoxon signed ranks test

## Discussion

The addition of RS to the habitual rural Malawian diet in preschool aged children changed the composition of the gut microbiota. The changes were modest and did not clearly produce gut bacteria thought to be anti-inflammatory. Fecal SCFA composition was altered after RS, but markers of gut inflammation were not decreased. Interestingly, fecal calprotectin was increased after adding RS to the diet and the bacterial microbiome showed increased expression in the lipopolysaccharide synthesis pathway, both of these findings suggesting subclinical gut inflammation. However, taken together, these data do not support the hypothesis that dietary RS reduces EED in this population.

This study was one of the first attempts to use prebiotics to improve gut health in sub-Saharan Africa. While compliance was excellent, changes in the microbiome did not increase SCFA or reduce fecal host inflammatory transcripts. The complexity and stability of the microbiome seems to be such that it is unlikely to be altered in a substantial manner with a singular dietary additive such as 8.5 g/day of RS, at least for a short term.

Limitations of this study are that fecal sampling on either the habitual diet or after the addition of RS were made at a single point in time. Perhaps a more complete picture of the changes could be seen for a series of samples collected from each subject. No placebo arm was included, so that any temporal changes that occurred during the 4-week study were not controlled for natural changes in the gut microbial composition in this age group. A limitation of the data analyses was that the microbiome before and after consumption of RS was compared with LEfSe, a software that does not account for the paired nature of the specimens. This results in a loss of statistical power to detect differences.

The genus composition of the microbiota of these rural African children on their habitual diet was dominated by *Prevotella*, manifesting a P-type community structure, which has been seen in populations that consume large amounts of dietary RS [20]. RS is particularly a good nutrient source for *Bacteroidetes*, with its ‘Sus-like systems’ to degrade complex carbohydrates with their multiple glycan branches [21]. *Bacteroidetes* was the dominant phylum we observed before and after our intervention. Our primary finding that the addition of dietary RS type 2 to the established P-type community of microbiota reduced diversity demonstrates the influence of the existing microbiota community on the effect of a prebiotic intervention.

In previous studies, ingestion of RS was accompanied by increased numbers of *Ruminococcus bromii*, *Parabacteroides distasonis*, *and Eubacterium rectale* in the gut, a finding that was not seen in our work [22–25]. This speaks to the complexity of the ecosystem within the human gut and suggests that changes in the populations of certain species are contingent upon multiple factors.

The amount of animal source macronutrients provided by the intervention was quite small, about 4 g/day, as the doughnuts were primarily composed of corn flour and soy oil. These animal source nutrients are likely to be almost completely absorbed in the small bowel and have little effect on the microbiota.

A previous study using the same RS at a similar dose in 4-year-old Indian children in Vellore for several days increased fecal acetate, propionate, and butyrate, and the authors concluded that the benefits of SCFA on colonic mucosa were likely conferred [26]. Feeding dietary sources of RS to healthy Australian adults was also associated with an increase in fecal SCFA [27]. We cannot be sure that SCFA were not produced in greater quantities after these Malawian children ingested RS, as the SCFA could have been absorbed. Our intervention was much longer than that in the Indian children, and the Malawian children had more severe stunting and likely harbored different communities of microbiota, which might account for the observed differences from the Indian children and Australian adults.

Out of more than 1600 metabolites putatively identified by mass spectrometry, just six were noted to be differentially expressed before and after the consumption of RS. It seems unlikely that these were directly administered in the intervention doughnut, as most of the doughnut was made from habitual foods and these molecules are not known to be by-products formed during the cooking process. Thus, we suspect that these molecules might be produced by microbiotia whose population was supported by the RS. PCA analyses clearly show a different population of metabolites after consumption of RS as well (Additional file 1: Figure S3). Studies of individual bacterial species introduced into germ-free mice show that rather a given taxa of microbes giving rise to an increase or decrease in a SCFA or a metabolite, changes in the metabolome are the result of the microbiota collectively [28, 29].

The increases in fecal calprotectin were modest but consistent after RS administration had more calprotectin. A normal fecal calprotectin concentration in children is <50 μg/g, while clinical colitis is associated with concentrations >1000 μg/g, and asymptomatic inflammatory bowel disease with concentrations 100–500 μg/g [30]. While the increases in calprotectin suggest more inflammation after RS starch administration, this increase was not accompanied by any clinical manifestations.

The reduction in the Shannon H index suggests that RS reduces diversity in the microbiota. The gut microbiome naturally becomes both more diverse and stable over the first 3 years of life [31, 32]. A loss of diversity in the gut microbiota has also been associated a greater risk of malnutrition [33].

After RS treatment, the LEfSe analysis showed an increase in bacterial iron complex outer membrane receptor protein, which would be expected with a decrease in luminal iron. Because RS increases iron absorption in piglets by decreasing the amount of iron available to the gut bacteria [34], this increase of bacterial iron complex outer membrane receptor protein could reflect the effort of the gut bacteria to cope with the decrease in iron available. Additionally, the upregulation of folate biosynthesis following RS treatment is consistent with the increased amounts of *Faecalibacterium* and *Prevotella* in the gut microbiota, as *Faecalibacterium prausnitzii* and *Prevotella ruminicola* are involved in folate biosynthesis.

## Conclusions

RS does not confer physiologically meaningful changes on gut biology, or gut microbial content, after short course treatment. Our data do not preclude the potential use of other prebiotics, but provide no data in support of using RS to improve gut health in this sub-Saharan childhood population.

## Methods

### Study design and population

Eighteen 3–5-year-old stunted children (8 boys and 10 girls) were selected from the rural village of Masika in the Machinga District in Malawi for the study. The children lived in subsistence farming families in unelectrified mud huts with grass roofs, without access to clean water. Stunting was defined as height-for-age Z score <−2. The children consumed a plant based, predominantly maize diet, with an animal source food <2/month. The 18 children studied were on average 44 ± 6.7 months old, with weight-for-height Z score 0.01 ± 0.7, and height-for-age Z score −3.2 ± 0.7 [13].

Fecal samples were collected 1 day before initiating dietary RS and 28 days after the RS was begun. Each fecal sample was snap frozen in liquid nitrogen within minutes of donation and kept at −80 °C until processing.

The study was approved by the Institutional Review Board from Washington University School of Medicine in St. Louis and the Research Ethical Committee of University of Malawi College of Medicine.

### Intervention

The free-living Malawian children enrolled in the study consumed about 8.5 g of RS in addition to their habitual Malawian diet, using locally produced doughnuts, known as mandasis, for 4 weeks [13]. Mandasis were typically consumed once per week by these children in their habitual diet. Mandasis for the study were made of white wheat flour, whole fluid milk, eggs, baking powder, salt, sugar, and RS (Hylon VII, HAMS 70 % amylose, National Starch and Chemical Company, Bridgewater, New Jersey) and fried in soybean oil. Each mandasi was 40 g and provided approximately 150 kcal, of which had 4 % protein, 26 % fat, 49 % digestible carbohydrate, and 10 % resistant starch. A study aid delivered the mandasis twice daily to each participant and observed the consumption of the doughnut.

### Isolation and characterization of microbiota

Approximately 100 mg of stool was resuspended in 0.5 ml stool lysis buffer (Buffer ATL, Qiagen, Valencia, California) and disrupted with 2.3 mm-zirconium/silica beads (Research Products International Corporation, Mount Prospect, Illinois) in the FastPrep-24 bead beater (MP Biomedicals, Santa Ana, California) for 2 min with a speed of 6.5 m/s twice. The tubes were centrifuged and 50 μl proteinase K solution (25 mg/ml, Qiagen, Valencia, California) was added. The tubes were incubated in a water bath at 56 °C for 30 min. Then, 20 μl RNase A (20 μg/μl) was added, mixed, vortexed, and briefly centrifuged before adding 1 ml lysis buffer Easy Mag. The samples were processed using the NucliSENS easyMAG “Specific A” (bioMérieux, Durham, NC) following the manufacturer’s instructions. Of the total nucleic acids, 110 μl were collected and 50 μl (ca. 50–100 ng/μl) were submitted to The Genome Institute at Washington University in St Louis.

The V1–V3 hypervariable regions of the 16S rRNA gene were amplified by PCR using the 27F and 534R primers “AGAGTTTGATCMTGGCTCAG” and “ATTACCGCGGCTGCTGG.” The V3–V5 hypervariable regions were amplified with 357F and 926R primers, “CCTACGGGAGGCAGCAG” and “CCGTCAATTCMTTTRAGT” used by the Human Microbiome Project [35]. The average read depths obtained with the V1–V3 and V3–V5 primers are provided in Additional file 1: Tables S1 and S2. The degree of overlap for the genera captured by the V1–V3 and V3–V5 regions are shown in Additional file 1: Figure S1. The PCR products were purified and sequenced at the Genome Institute at Washington University using the Genome Sequencer Titanium FLX (Roche Diagnostics, Indianapolis, Indiana).

Sample sequences were binned by locating and removing their tags in flow space, with one mismatch allowed (sff file). Fasta and qual files were then generated, and the primers were removed from the 3′ end of the sequence, allowing one mismatch in addition to primer degeneracies. Low quality bases were removed using Mothur software with the parameter trim.seqs(qaverage = 35) [36]. Sequences less than 200 bases were removed, and taxonomic calls were generated for each read using the Ribosomal Database Project Naïve Bayesian Classifier version 2.5 with training set 9 [37, 38]. Chimeric sequences were identified and removed using ChimeraSlayer with default parameters [37].

To analyze the diversity at various taxonomic levels, RDP-generated taxonomic calls were analyzed using in-house Perl script to generate sample vs. taxonomy matrices, where a 0.5 confidence level was required to accept a call at each taxonomic level and reads with <0.5 confidence at a level, for example genus, were considered unclassified at the family level. Because different samples yielded different sequence depth, read subsampling, or rarefaction, a depth of 1000 reads was done using the Vegan package in R [39].

### Microbiome analysis

Whole genome shotgun libraries were generated and sequenced on the Illumina HiSeq 2000 platform [40]. The reads generated were 100 bp in length. Illumina genomic DNA sequence for 37 WGS samples were retrieved from the Laboratory Information Management System database and subject to human contaminant screening using BMTagger. The non-human reads were then filtered to remove redundancy (using Picard’s Estimate Library Complexity method (release 1.27)), low quality reads were trimmed using the TrimBWAStyle.pl script, which applies the quality trimming logic used by Burrows-Wheeler Aligner, and finally low complexity reads as detected by the DUST program were removed. DUST masks low quality sequence that it finds, and reads were discarded anytime fewer than 60 unmasked bases remained after applying DUST.

These cleaned reads were then mapped to the latest publically available release of the Kyoto Encyclopedia of Genes and Genomes (KEGG) genes database (v58) using the MBLASTX program with default parameters [41]. Results of this mapping were used as input to The Human Microbiome Project Unified Metabolic Analysis Network program [42], to obtain pathway and KEGG identifier abundances. These abundances were finally fed into the LEfSe [43] and used to calculate enriched pathways and/or KEGG identifiers between defined groupings of samples.

### Determination of SCFA

Fecal SCFA content was determined by gas chromatography/mass spectrometry (GC/MS) with stable isotope labeled internal standards [44]. Samples were thawed, weighed, and 50 nmol each internal standard was added (acetate ^13^C_2_;d_3_, propionate-d_5_ and butyrate ^13^C_4_). The samples were diluted with 1 ml 0.1 mM HCl and extracted into 500 μl t-butylmethyl ether. A 150-μl aliquot of the ether layer was introduced into an autosampler insert, and 50 μl *N*-methyl-*N*-*tert*-butyldimethylsilyltrifluoroacetamide was added. Derivatized samples were analyzed on an Agilent 7890A GC interfaced to an Agilent 5975C MS. The GC column used for the study was a HP-5MS (30 m, 0.25 mm i.d., 0.25 μm film coating, P.J. Cobert St. Louis, Missouri). A linear temperature gradient was used. The initial temperature of 60 °C was held for 3 min and increased to 300 °C at 15 °C/min before holding at 300 °C for 2 min. The samples were subjected to electron ionization and the source temperature, electron energy, and emission current were 230 °C, 70 eV, and 300 μA, respectively. The injector and transfer line temperatures were 250 °C.

### GC/MS-based fecal metabolomic profiling

Of fecal sample, 13–35 mg was suspended in 400 μl isopropanol:CH3CN:0.1 % FA (3:3:2), vortexed, and stored overnight at −20 °C. These extracts were centrifuged, 300 μl were transferred into an autosampler vial, taken to dryness under N_2_, and chemically derivatized using *N*-Methyl-*N*-(trimethylsilyl)_trifluoroacetamide/CH3CN (1:3) for 2 h at room temperature. Quality control samples were then prepared by pooling 10 μl from each sample and injected after every eight samples. Derivatized samples were analyzed as described above with an initial temperature of 80 °C for 2 min and a 10 °C/min ramp.

Identification and comparison of the metabolites was made using the Mass Profiler Professional software (Agilent Technologies, Santa Clara, CA). Software modules were used to display and statistically compare the orthogonally transformed complex data matrix using a principal component analysis (PCA) and a 3-dimensional plot. This involved converting the observed mass spectra-related variable matrix (e.g., signal intensity, mass/charge, retention time, before vs after RS) into a set of linearly uncorrelated variables referred to as principal components where the first principal component accounts for the greatest amount of variance in the matrix, the second principal component the second most variance, and the third principal component the third most variance, such that these principal components represent the eigenvectors of the covariance matrix. This reduces the dimensionality of the complex data matrix and reveals the structure of the variable matrix in a manner that displays and considers the variance in the data. The resulting 3-dimensional PCA plot display clusters of data points where the amount of overlap between the clusters is a reflection of the degree of similarity or diversity between the data sets (before versus after RS).

### Detection of fecal inflammatory transcripts and proteins

Nucleic acids from 200 mg human stools samples of 18 children were extracted using the NucliSens easyMAG system (bioMérieux, Durham, NC) and eluted into 110 μl final volume. Ten different fecal human mRNA transcripts were quantified using digital droplet PCR with the QX100 Droplet Digital system (BioRad, Hercules, California) and analyzed in a droplet reader as previously described [45].

The calprotectin protein was measured in the fecal samples by using a PhiCAL Fecal Calprotectin Immuno assay kit (Calpro AS, Norway) and expressed in μg calprotectin/g stool.

### Statistical analyses

Subjects served as their own controls. For the microbiota, SCFA, host inflammatory transcripts, and proteins, comparisons were made for each of the 18 children between the habitual diet, and the habitual diet + RS were made using a Wilcoxon Signed rank test by SPSS ver. 21.0 (IBM Corp, Armond, NY) statistical software. For fecal metabolites, the ratio of peak area after RS to the habitual diet was calculated and the distribution of these ratios was tested for significance by calculating the 95 % confidence interval using GraphPad Quick Calcs (La Jolla, California). For all comparisons, a *P* < 0.05 was considered significant. All *p* values were two-tailed.

## Availabilty of supporting data

The 16S sequences used in this paper is included in an Additional file 2.

## Additional files

Additional file 1: Table S1. Number of 16S rRNA and wide genome sequences generated per sample. This table shows the number of raw (unprocessed) reads and the number of reads remaining after analytical processing of 18 samples from children fed with the habitual diet and plus the addition of RS. **Table S2** Comparison of the number of reads obtained by V1–V3 and V3–V5 sequencing. **Figure S1** Venn diagram showing the degree of overlap for genera captured with the V1–V3 and V3–V5 regions. While the majority of taxa are captured by both sets of primers, the fact that a significant subset were captured by only one primer set made the use of both sets more valuable. **Figure S2** LEfse rank plot of differentially abundant genes in gut microbiomes initial samples vs. final samples. LDA scores were given for different abundance of genes before (habitual diet: green) and after the resistant starch was added to the habitual diet (habitual diet + RS: red). **Figure S3** Principal component analysis (PCA) plot showing the differential clustering of metabolites in fecal samples collected before (blue-habitual diet) and after (red-habitual diet plus RS) the diet supplementation with RS. Brown circles represent replicate analyses of the pooled quality control (QC) fecal samples. The tight clustering of these QC replicate analyses indicates the high reproducibility and low amount of drift associated with the GC/MS-based fecal metabolomic profiling analyses conducted over several hours while the individual fecal extracts are analyzed at the same time, under identical GC/MS conditions, and on the same GC/MS instrument. Additional file 2:
**16S rRNA raw reads analyzed in this study.**

## Abbreviations

EED: environmental enteric dysfunction
KEGG: Kyoto Encyclopedia of Genes and Genomes
RS: resistant starch
SCFA: short chain fatty acids
