# Supplementary material for: The effect of dietary resistant starch type 2 on the microbiota and markers of gut inflammation in rural Malawi children
Source: Microbiome. 2015 Sep 3;3:37. doi: 10.1186/s40168-015-0102-9 (PMC4558878; doi:10.1186/s40168-015-0102-9)

**Table S1.** Number of 16S rRNA and Wide Genome Sequences generated per sample. This table shows the number of raw (unprocessed) reads and the number of reads remaining after analytical processing of 18 samples from children fed with the habitual diet and plus the addition of RS.

| Sample | 16S  Raw reads | 16S  Processed reads | WGS  Raw reads | WGS  Processed reads |
| --- | --- | --- | --- | --- |
| 1-Habitual diet + RS | 23394 | 10178 | 59972804 | 57553091 |
| 1-Habitual diet | 26843 | 12454 | 81142976 | 77930772 |
| 10-Habitual diet + RS | 7609 | 2432 | 66749696 | 63606275 |
| 10-Habitual diet | 23505 | 3166 | 88327146 | 83875032 |
| 11-Habitual diet + RS | 21706 | 4013 | 66799364 | 55827755 |
| 11-Habitual diet | 6179 | 2427 | 57758720 | 55989508 |
| 12-Habitual diet + RS | 23997 | 11091 | 86694566 | 83419038 |
| 12-Habitual diet | 19493 | 7913 | 86546104 | 82763383 |
| 13-Habitual diet + RS | 20582 | 9822 | 63719632 | 32290308 |
| 13-Habitual diet | 18267 | 7907 | 53038048 | 50717179 |
| 14-Habitual diet + RS | 16568 | 7353 | 60586322 | 58014951 |
| 14-Habitual diet | 22399 | 9614 | 44303156 | 42855352 |
| 15-Habitual diet + RS | 16654 | 7110 | 64685132 | 62260902 |
| 15-Habitual diet | 26739 | 11767 | 70064590 | 67872672 |
| 16-Habitual diet + RS | 15542 | 6074 | 78052286 | 68286852 |
| 16-Habitual diet | 21439 | 9251 | 52316668 | 50179787 |
| 17-Habitual diet + RS | 16789 | 6597 | 75984110 | 72912521 |
| 17-Habitual diet | 26850 | 11388 | 66680578 | 63966340 |
| 19-Habitual diet + RS | 20081 | 9039 | 71923114 | 68590855 |
| 19-Habitual diet | 31193 | 15090 | 66961080 | 64441495 |
| 2-Habitual diet + RS | 60510 | 31518 | 45017938 | 43555978 |
| 2-Habitual diet | 25078 | 10131 | 44841504 | 43445905 |
| 20-Habitual diet + RS | 23718 | 10486 | 27762094 | 26898029 |
| 20-Habitual diet | 20905 | 8941 | 73666034 | 70576237 |
| 3-Habitual diet + RS | 12577 | 5983 | 93683200 | 89724336 |
| 3-Habitual diet | 24671 | 10409 | 60288892 | 58330277 |
| 4-Habitual diet + RS | 17652 | 7268 | 97604256 | 93246922 |
| 4-Habitual diet | 32864 | 14043 | 66098988 | 64003636 |
| 5-Habitual diet + RS | 22441 | 10704 | 66133196 | 63366012 |
| 5-Habitual diet | 16236 | 7229 | 60820688 | 58541559 |
| 6-Habitual diet + RS | 10358 | 4554 | 76949718 | 73824058 |
| 6-Habitual diet | 28451 | 11812 | 45084442 | 43660948 |
| 7-Habitual diet + RS | 28514 | 13222 | 75444412 | 72118858 |
| 7-Habitual diet | 17151 | 7007 | 36624288 | 35539941 |
| 8-Habitual diet + RS | 24345 | 4385 | 68298134 | 65675186 |
| 8-Habitual diet | 5042 | 1501 | 62222912 | 59511292 |

**Table S2.** Comparison of the number of reads obtained by V1-V3 and V3-V5 sequencing.

| **Region** | **Total Samples** | **Paired Samples** | **Failed Samples (<1000 reads)** | **Average**  **# reads per sample** |
| --- | --- | --- | --- | --- |
| **V1-V3** | **47** | **32** | **5** | **5639** |
| **V3-V5** | **47** | **35** | **0** | **3489** |

**Figure S1**. Venn diagram showing the degree of overlap for genera captured with the V1-V3 and V3-V5 regions. While the majority of taxa are captured by both sets of primers, the fact that a significant subset were captured by only one primer set made the use of both sets more valuable.


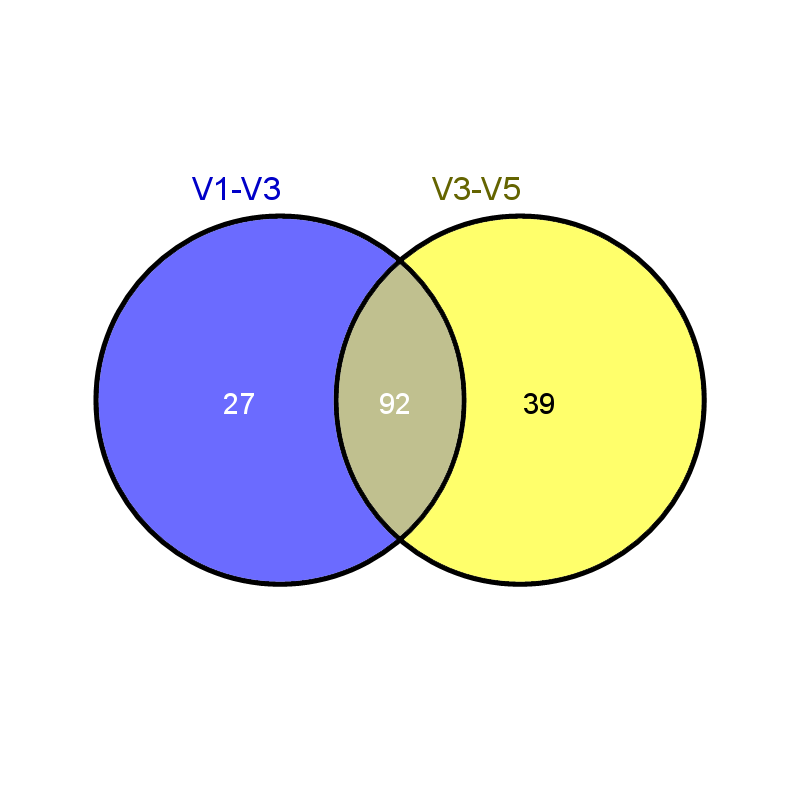


**Figure S2**. LEfse rank plot of differentially abundant genes in gut microbiomes initial samples vs. final samples. LDA scores were given for different abundance of genes before (habitual diet:GREEN) and after the resistant starch was added to the habitual diet (habitual diet + RS:RED).


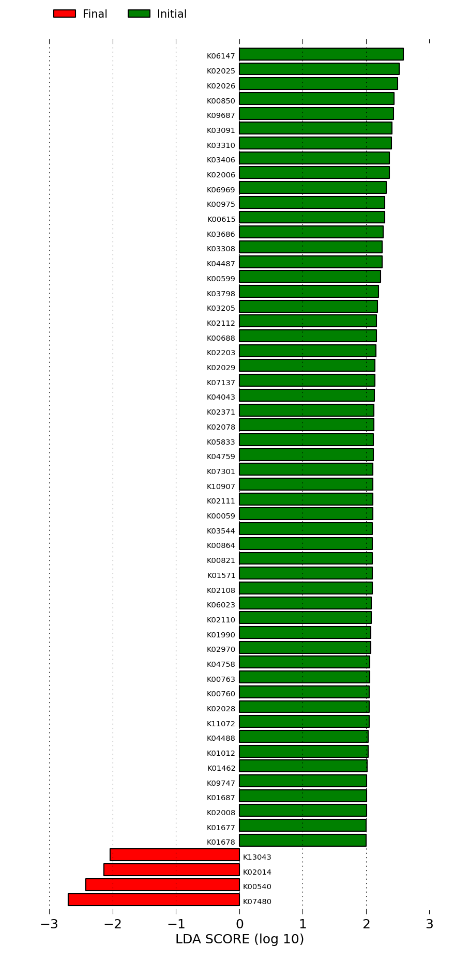


Habitual Diet + RS

Habitual Diet


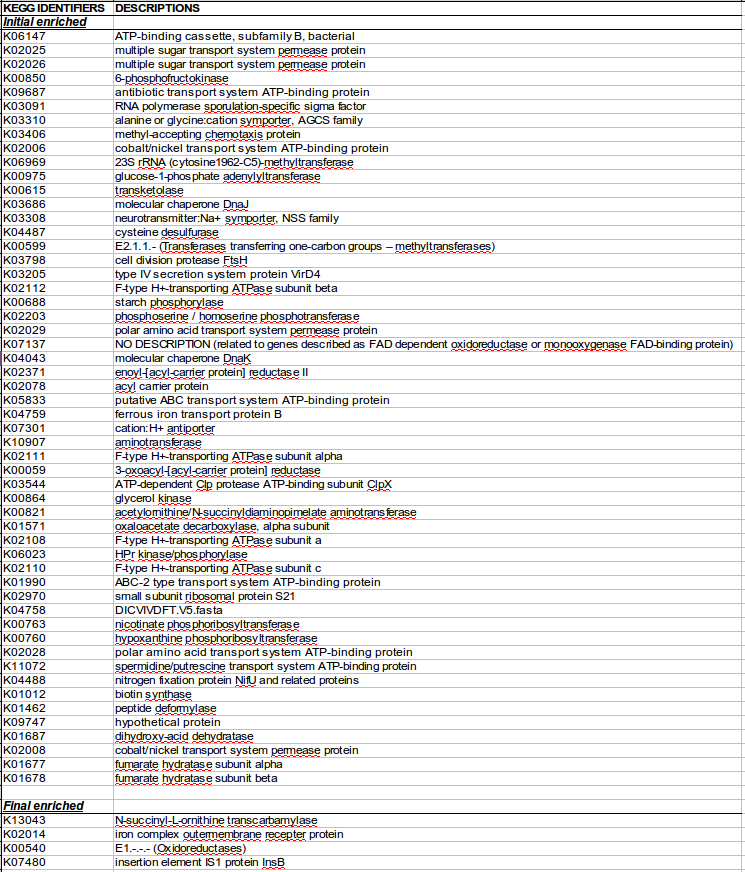


**Figure S3.** Principal Component Analysis (PCA) plot showing the differential clustering of metabolites in fecal samples collected before (blue - habitual diet) and after (red - habitual diet plus RS) the diet supplementation with RS. Brown circles represent replicate analyses of the pooled quality control (QC) fecal samples. The tight clustering of these QC replicate analyses indicates the high reproducibility and low amount of drift associated with the GC/MS-based fecal metabolomic profiling analyses conducted over several hours while the individual fecal extracts are analyzed at the same time, under identical GC/MS conditions, and on the same GC/MS instrument.


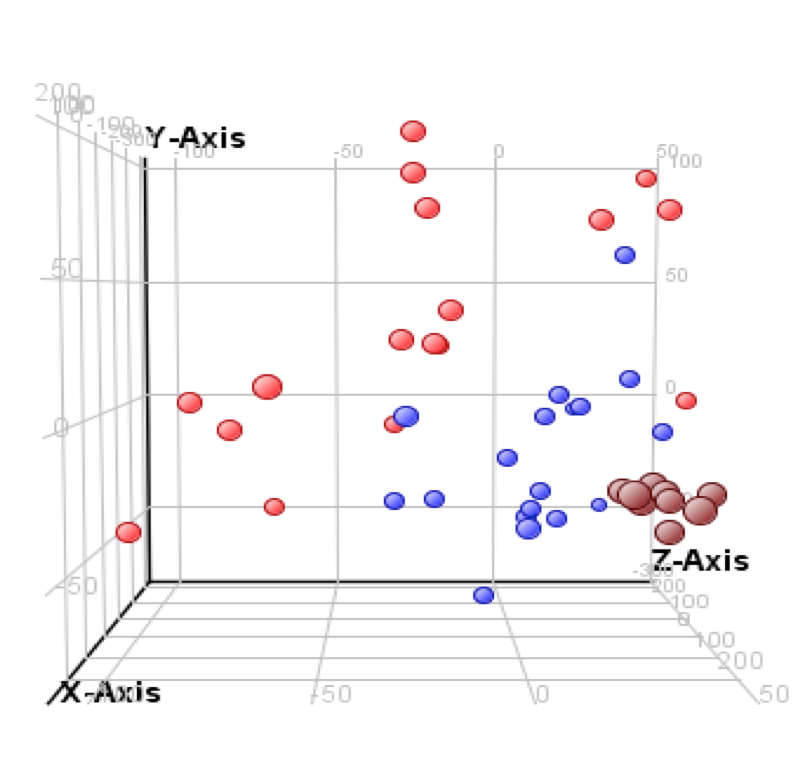

Supplement: Additional file 1: Table S1. — Number of 16S rRNA and wide genome sequences generated per sample. This table shows the number of raw (unprocessed) reads and the number of reads remaining after analytical processing of 18 samples from children fed with the habitual diet and plus the addition of RS. Table S2 Comparison of the number of reads obtained by V1–V3 and V3–V5 sequencing. Figure S1 Venn diagram showing the degree of overlap for genera captured with the V1–V3 and V3–V5 regions. While the majority of taxa are captured by both sets of primers, the fact that a significant subset were captured by only one primer set made the use of both sets more valuable. Figure S2 LEfse rank plot of differentially abundant genes in gut microbiomes initial samples vs. final samples. LDA scores were given for different abundance of genes before (habitual diet: green) and after the resistant starch was added to the habitual diet (habitual diet + RS: red). Figure S3 Principal component analysis (PCA) plot showing the differential clustering of metabolites in fecal samples collected before (blue-habitual diet) and after (red-habitual diet plus RS) the diet supplementation with RS. Brown circles represent replicate analyses of the pooled quality control (QC) fecal samples. The tight clustering of these QC replicate analyses indicates the high reproducibility and low amount of drift associated with the GC/MS-based fecal metabolomic profiling analyses conducted over several hours while the individual fecal extracts are analyzed at the same time, under identical GC/MS conditions, and on the same GC/MS instrument. [file 40168_2015_102_MOESM1_ESM.docx]
